# Developmental profiles of SUMOylation pathway proteins in rat cerebrum and cerebellum

**DOI:** 10.1371/journal.pone.0212857

**Published:** 2019-02-22

**Authors:** Fernando Josa-Prado, Jia Luo, Philip Rubin, Jeremy M. Henley, Kevin A. Wilkinson

**Affiliations:** 1 Universidad Alfonso X el Sabio, Avda, de la Universidad, Madrid, España; 2 School of Biochemistry, Medical Sciences Building, University of Bristol, University Walk, Bristol, United Kingdom; Instituto Cajal-CSIC, SPAIN

## Abstract

Protein SUMOylation regulates multiple processes involved in the differentiation and maturation of cells and tissues during development. Despite this, relatively little is known about the spatial and temporal regulation of proteins that mediate SUMOylation and deSUMOylation in the CNS. Here we monitor the expression of key SUMO pathway proteins and levels of substrate protein SUMOylation in the forebrain and cerebellum of Wistar rats during development. Overall, the SUMOylation machinery is more highly-expressed at E18 and decreases thereafter, as previously described. All of the proteins investigated are less abundant in adult than in embryonic brain. Furthermore, we show for first time that the profiles differ between cerebellum and cerebrum, indicating differential regional regulation of some of the proteins analysed. These data provide further basic observation that may open a new perspective of research about the role of SUMOylation in the development of different brain regions.

## Introduction

SUMOylation is the covalent attachment of a 97-residue protein, SUMO (**S**mall **U**biquitin-related **MO**difier), to lysine residues on target proteins. SUMOylation is best characterised for modifying nuclear proteins involved in genome integrity, nuclear structure and transcription [1, 2] but it is now clear that SUMOylation is also important for extranuclear signal transduction, trafficking and modification of cytosolic and integral membrane proteins. Several hundred SUMOylation substrates have been validated and many more candidate substrates have been identified by proteomic studies [3–5].

There are three SUMO paralogues (SUMO-1-3) in vertebrates. SUMO-2 and SUMO-3 are identical except for three residues, but share only ~50% sequence identity with SUMO-1. While some substrates can be modified by both SUMO-1 and SUMO-2/3, SUMO proteins are functionally heterogeneous and show distinct patterns of conjugation under both resting conditions and in response to cell stress. For example, under resting conditions there is very little unconjugated SUMO-1 whereas there is a large free pool of SUMO-2/3 [6]. However, in response to a variety of stressors, SUMO-2/3 conjugation is dramatically increased while SUMO-1 conjugation is relatively unchanged [6–13]. The functional consequences of SUMO attachment are in many cases poorly understood and can vary greatly depending on the substrate.

The SUMOylation state of substrate proteins is a dynamic balance between conjugation and deconjugation. Briefly, inactive precursor SUMO is matured by SUMO-specific proteases (SENPs) to expose a C-terminal diglycine motif, which is activated by an ATP-dependent E1 enzyme, formed by a heterodimer of SAE1 and SAE2 [14]. E1 passes the activated SUMO onto the specific and unique SUMO conjugating E2 enzyme Ubc9 via a transesterification reaction [15, 16]. Ubc9, often in conjunction with a growing number of identified E3 ligase enzymes, then catalyses SUMOylation of the substrate.

SUMO is removed from substrates by the isopeptidase activity of the SENPs, the same enzymes required for pro-SUMO maturation. There are six mammalian SENPs (SENP1-3 and SENP5-7; for reviews, see [17–19]). SENP1 and SENP2 have a broad specificity for SUMO-1 and SUMO-2/3 and are involved in both maturation and deconjugation while SENP3 and SENP5 favour SUMO-2/3 over SUMO-1. They selectively remove SUMO-2/3 from substrate proteins and do not play a role in SUMO maturation. SENP6 and 7 are primarily involved in the editing of poly-SUMO chains. Furthermore, three proteins distinct from the SENP family have also been reported to function as deSUMOylating enzymes [19].

SUMOylation is involved in multiple signalling cascades in neurons and is strongly implicated in many neurological and neurodegenerative diseases including Huntington’s disease, Alzheimer’s disease, Parkinson’s disease and stroke (for reviews see [20–23]). Importantly, protein modification by SUMO2/3 is neuroprotective against metabolic cell stress [24, 25, 26]. Furthermore, we have shown that SUMOylation is a key modulator of synaptic transmission and plasticity via direct modulation of kainate receptors [27–29] and presynaptic proteins such as Syntaxin 1A [30] and Synapsin Ia [31] and indirect regulation of AMPAR trafficking in synaptic scaling [32] and LTP [29]. Recently, it has been shown that SUMOylation might be important in neuronal development through modification of the transcription factors FOXP1 and MEF2A [33, 34].

Here we set out to define the developmental profiles of SUMO machinery proteins and the extent of total protein SUMOylation in fractions of rat cerebrum and cerebellum. The aim of this work was to provide a framework for defining how protein conjugation by SUMO-1 and SUMO-2/3 changes during brain development.

## Materials and methods

### Brain lysates

Lysates were prepared from crude cerebrum and cerebellum from Wistar rats at the indicated ages. Animals were bred by an in-house animal unit and sacrificed by cervical dislocation in accordance with UK Home Office Schedule 1 guidelines. All procedures were approved by the Animal Welfare and Ethics Review Body (AWERB) at the University of Bristol (approval reference number UIN UB/18/004). In all cases brains from at least 3 separate rats were used. Seven developmental stages were investigated: embryonic day 18 (E18) and postnatal ages (in days) P1, P3, P7, P14, P21 and adult (pregnant rat from 12 weeks onwards). Female rats were used for all samples except E18, which were not sexed. For E18 more embryos were sacrificed to get sufficient tissue and samples were taken from different litters. Following sacrifice, brains were immediately taken and the cerebellum and cerebrum separated. These were then homogenised in 25mM HEPES, 150 mM NaCl, 1x protease inhibitor cocktail (Complete tablets, Roche), pH 7.5 supplemented with 20 mM NEM (to avoid deSUMOylation after cell lysis).

The volume of buffer used was proportional to the wet weight of the tissue (10 ml of buffer per gram of tissue) and samples were homogenized with a Teflon and glass homogeniser (around 10 passes). The homogenate was then collected and solubilised by the addition of 1% Triton X-100 and 0.1% SDS (final concentration) and incubation on a rotating wheel at 4°C for 40 minutes. Cellular debris was separated by centrifugation at 16,000g for 10 minutes and the insoluble pellet discarded. The total protein concentration in the soluble fraction was measured by Bradford assay and samples mixed with 6x Laemmli buffer, boiled and diluted to a concentration of 1 mg/ml.

### Subcellular fractionation

Fractions were obtained by differential centrifugation [35]. Briefly, tissue from either cerebrum or cerebellum of adult rat was homogenised in 10 ml/g of tissue in Tris-sucrose buffer (10mM Tris-HCl, pH7.4, 0.32M sucrose, 20mM NEM and protease inhibitors). The crude nuclear fraction (N) was obtained by centrifugation at 412g for 10 minutes and this P1 fraction was washed twice in the same buffer. The P1 supernatant (S1) was centrifuged at 10,000g for 20 minutes to obtain a crude synaptosomal fraction (P2). This was resuspended and loaded onto a discontinuous 0.32, 0.8, 1.2M sucrose gradient and centrifuged at 53,000g for 2 hours. The final synaptosomal (Sy) fraction was collected from the interphase between the 0.8M and 1.2M sucrose layers. The cytosolic fraction (S3) was obtained by centrifuging S2 at 20,000g for 1 hour. All fractions were solubilised in 0.1% SDS and 1% Triton X-100. Protein concentration was measured by Bradford assay and samples equalized to the same protein concentration in Laemmli buffer for SDS-PAGE.

### Western blotting

Samples from each age point were subjected to SDS-PAGE on 8–15% polyacrylamide gels, depending on the protein being detected. For SUMO blots, gradient gels (4–20%) were used. Separated proteins were transferred to nitrocellulose membrane and immunoblotted. The primary antibodies used were: β-III-tubulin (1:5000, T8660), β-actin (1:2500, A5441), Syntaxin1A (1:5000, S0664) all from Sigma-Aldrich. PSD95 (1:1000, AB1596), GluA1 (1:1000, AB2263), NMADR1 (1:1000, AB9864) from Millipore. Aos1 (1:200, sc-46766), Uba2 (1:200, sc-376305), SUMO-1 (1:500, sc-5308) from Santa Cruz Biotechnology. Ubc9 (1:250, 610749) from BD Biosciences; PIAS1 (1:2000, 77231) from ABCAM; PIAS3 (1:1000, AP1245a) from Abgent; SENP3 (1:4000, D20A10) Cell Signaling Technology; SUMO-2/3 (1:100, Clone 8A2, produced in house) from DRHB. Luminescent or fluorescent signal was captured with a Li-COR Odyssey Fc system and quantified using Image Studio 2.1 provided by Li-COR. For SUMO quantification the whole lane was selected and quantified as well as individual bands with strong signal.

### Statistical analysis

1-way ANOVA with Newman-Keuls *post-hoc* test was used for analysis of the differences between time points in cerebrum or cerebellum compared to E18, which was designated as 100%. For determining differences between cerebrum and cerebellum in each time point for each protein two-way ANOVA with Bonferroni *post-hoc* test was used (* = p≤0.05, ** = p≤0.01, *** = p≤0.001).

## Results

In order to address how profiles of protein SUMOylation and SUMO pathway proteins are altered in cerebellum and cerebrum during development, we homogenised rat brains at various stages of development (embryonic day 18, and post-natal days 1, 3, 7, 14, 21 and adult). Importantly, due to their small size, the embryonic day 18 (E18) brains were not dissected into cerebrum and cerebellum, and instead we prepared whole homogenate from these brains. However, this approach had the added advantage of meaning that all cerebellum and cerebrum samples from the other time points were run against the same reference E18 samples, allowing direct comparison of changes in protein levels between the brain regions.

To validate our homogenate samples, we initially investigated the developmental profiles of several marker proteins (S1 and S2 Figs). Cytoskeletal proteins commonly used as loading controls, such as β-III-tubulin and β-actin, showed some developmental variation thus not being suitable as loading control (S1 Fig). Nonetheless, we loaded equal amounts of protein for each sample using Bradford assay to measure total protein concentration. We also monitored the profiles of several synaptic proteins: PSD95, syntaxin1A, GluA1 and NR1. Importantly, the profiles of PSD95 [36, 37], GluA1 [38] and NR1 [39–41] were consistent with previous literature, validating our samples (S2 Fig).

The samples show the expected changes and developmental trends in synaptic proteins, with respect to the literature, and are thus a reliable set of samples upon which to study the profile of proteins of the SUMOylation machinery, as well as global SUMOylation.

### Profiles of SUMOylation machinery proteins

**Fig 1:**
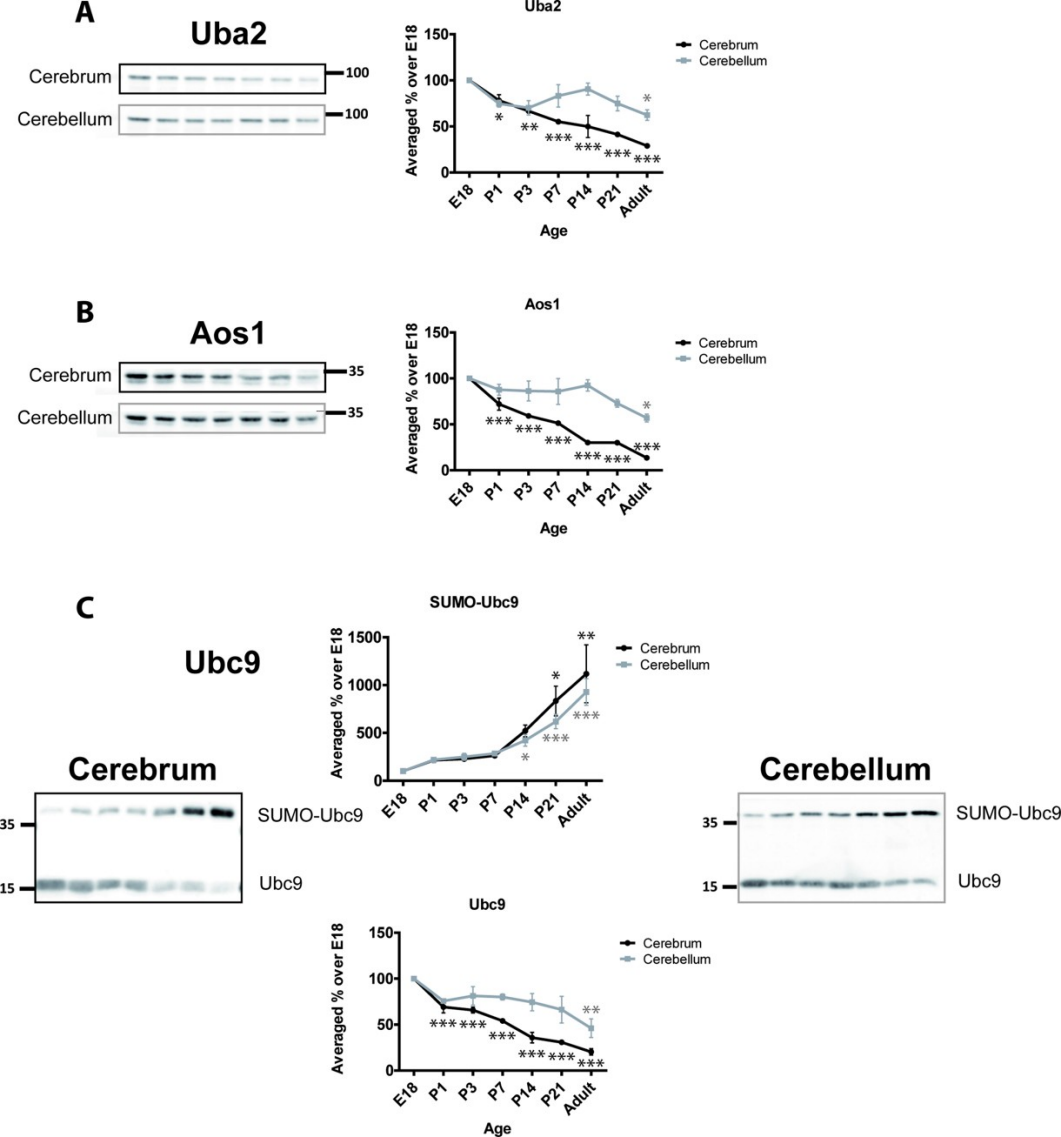
Developmental profiles of SUMO E1 and E2 enzymes. Upper panels, Representative immunoblots and quantification of the age-dependent profiles of E1 enzymes Uba2 and Aos1. Lower panels, SUMOylated and non-SUMOylated Ubc9 show inverse developmental profiles. Graphs show the levels of immunoreactivity at different ages expressed as a percentage of the levels present in E18 brain. (n = 3, * = p≤0.05, ** = p≤0.01, *** = p≤0.001).

We next determined the developmental profiles of various SUMO pathway proteins. The E1 ligase in the SUMOylation pathway comprises a heteromer of Uba2 and Aos1. In the cerebrum, Uba2 steadily and continuously decreases with age (Fig 1A). At P7 levels are significantly less than at from E18 (55.2% ± 1.8%) and in adult levels are 30.0% ± 0.8% of the E18 values. In cerebellum, Uba2 displays a slightly different profile with an initial decrease at P1 (74.7% ± 4.7%) followed by a stabilisation of levels until ~P21 followed by a further decrease in the adult (62.3% ± 5.7%; Fig 1A). As expected, since they act as a dimer, the profiles for Aos1 match those for Uba2 in both cerebrum and cerebellum. In cerebrum, Aos1 levels continuously decrease with age to 13.5% ± 1.2% of the E18 value in adult. In cerebellum, Aos1 is expressed at levels similar from E18 until P14 (92.4% ± 6.1%) and thereafter decreases to 57.0% ± 4.3% in adult (Fig 1B).

The sole SUMO E2 conjugating enzyme, Ubc9, has been previously reported to be SUMOylated [42, 43]. We detected a robust SUMOylated band for Ubc9 so we profiled and compared both SUMOylated and non-SUMOylated Ubc9 bands (Fig 1C). Unmodified Ubc9 decreases over development with 20.3% ± 3.5% of the E18 levels present in adult cerebrum. Similarly, in the cerebellum, unmodified Ubc9 levels decrease, albeit slightly less rapidly, to 46.1% ± 10.2% of the E18 level in adult.

In stark contrast, we observed age-dependent increases in SUMOylated Ubc9. In both cerebrum and cerebellum we observed an ~10-fold increase in SUMOylated Ubc9 in adult compared to E18. These observations are consistent with findings from another group that reported a similar increase in SUMOylated Ubc9 [43].

**Fig 2:**
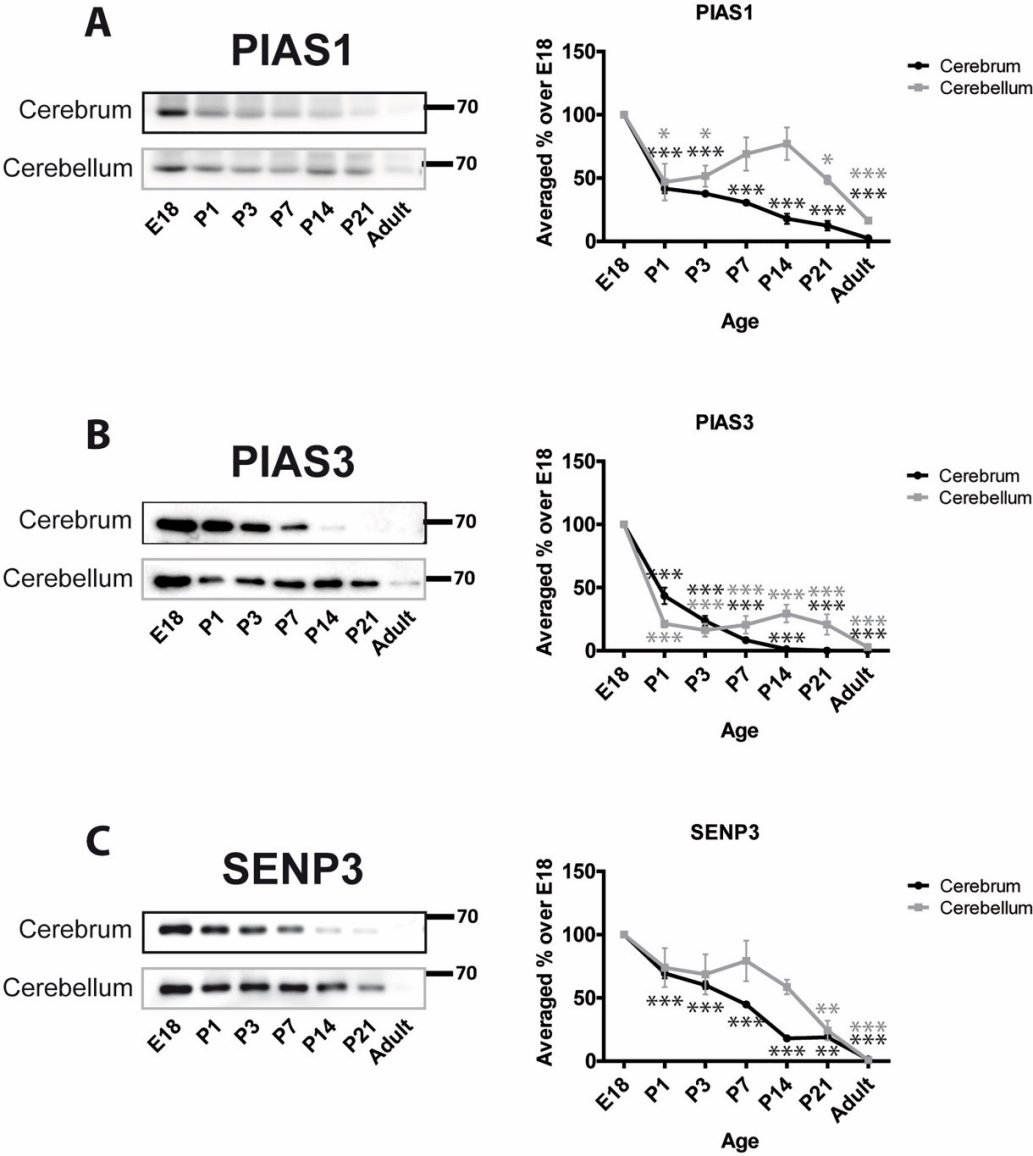
Developmental profiles of SUMO E3 ligases and the deSUMOylating enzyme SENP3. Representative blots for PIAS1, PIAS3 and SENP3 and graphs showing the quantified levels of immunoreactivity from cerebrum and cerebellum homogenates at the designated age points. Graphs show the levels of immunoreactivity at different ages expressed as a percentage of the levels present in E18 brain. (n = 3, * = p≤0.05, ** = p≤0.01, *** = p≤0.001).

SUMO E3 ligases can determine the specificity of substrate SUMOylation [3]. We monitored two proteins of the PIAS family of RING type E3 ligases, PIAS1 and PIAS3 [44]. Levels of PIAS1 decrease from E18 to P1 in cerebrum (41.6% ± 3.8%) and cerebellum (46.8% ± 14.5%). In cerebrum the decrease continues with very low levels of PIAS1 in adults compared to E18 (2.4% ± 1.3%; Fig 2A). However, in cerebellum PIAS1 levels showed a distinct profile. After the initial decrease between E18 and P1, PIAS1 levels stabilize/recover slightly before decreasing to 16.4% ± 1.5% in adult. The developmental profile of PIAS3 showed a similar pattern to PIAS1 but displayed an even greater initial decrease around birth. In cerebellum and cerebrum PIAS3 levels at P1 were 21.4% ± 2.9% and 43.4% ± 6.5%, respectively, of those at E18. PIAS3 levels in the cerebrum continue to decrease with age and are almost undetectable in adult (0.03% ± 0.016%). Similarly, in adult cerebellum PIAS3 levels decrease to 2.9% ± 1.1% of those at E18 (Fig 2B).

We also monitored levels of SENP3 as a representative of the SENP family of deSUMOylating proteins. The age-dependent profile was similar to those of the E3 ligases with a steady reduction in SENP3 levels from E18 to adult. At P21 SENP3 levels were 18.9% ± 2.6% and 24.3% ± 7.8% and in adult 1.4% ± 0.4% and 1.0% ± 0.2% of the E18 level in cerebrum and cerebellum, respectively. Interestingly, the decrease in cerebellum starts after P7 while in cerebrum is continuous from E18 (Fig 2C).

Overall, we can see that, in general, SUMO machinery proteins hold their levels higher in cerebellum than cerebrum, at least between P7 and P21, to decrease and be closer to each other by adult

### Age-dependent changes SUMO-1 and SUMO-2/3 conjugation

We assessed levels of SUMO conjugation using anti-SUMO-1 and anti-SUMO-2/3 antibodies. In both cases, we subjected the entire lane of the Western blot to densitometry to define the total amount of protein SUMOylation. In addition, two very prominent bands at 100 and 35 kDa in SUMO-1 blots, and 90 and 25 kDa in SUMO-2/3 blots were analysed separately and, because these bands make up a large proportion of the entire SUMO signal, they were subtracted from the ‘total’ SUMO values.

**Fig 3:**
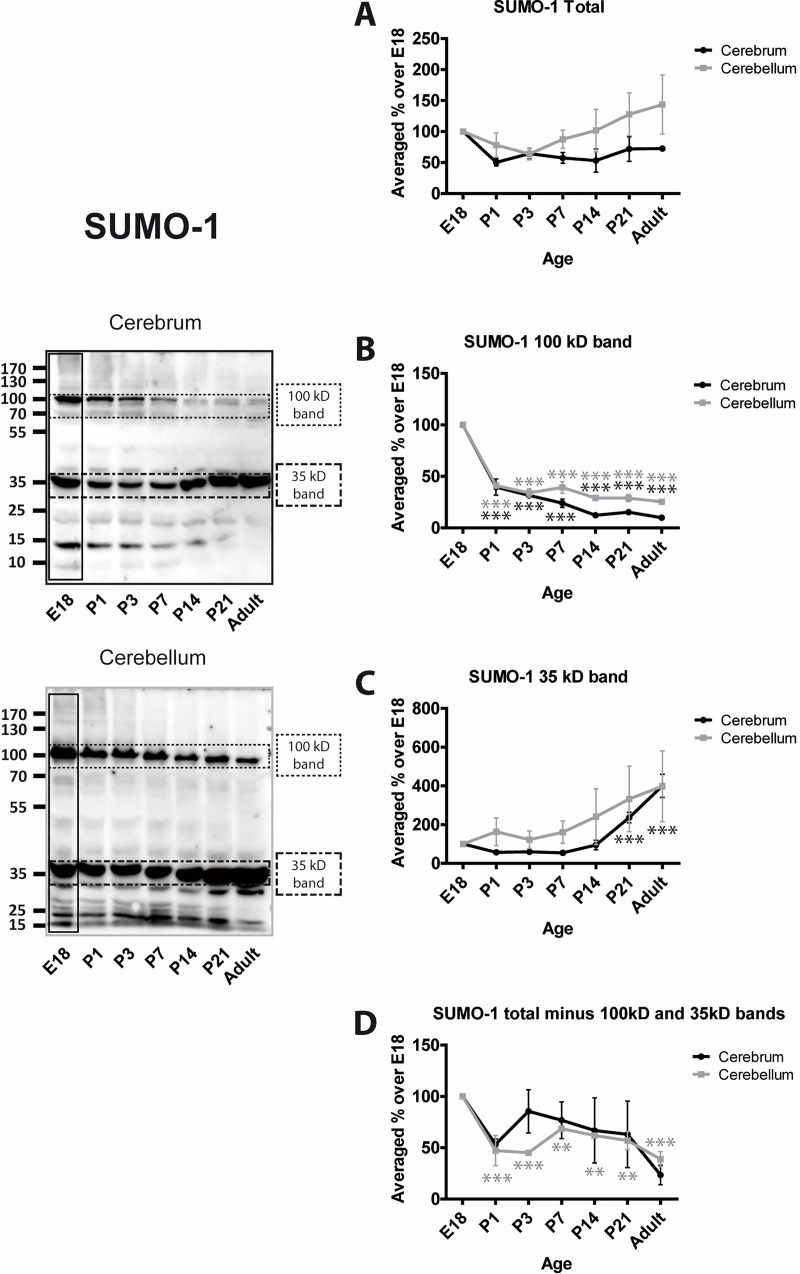
Levels of SUMO-1 conjugation to substrate proteins. Representative blots and quantification of the intensity of the whole SUMO-1 lane, the indicated bands and the whole lane minus the indicated bands. Total SUMO-1 immunoreactivity was quantified by taking the whole lanes (vertical black rectangle). Since the 100 kDa and 35 kDa bands are very intense and exhibit different profiles, they were subtracted from the total and the remaining signal plotted (Total (-) bands). This represents the level of SUMOylation of the other proteins in the sample. Graphs show the levels of immunoreactivity at different ages expressed as a percentage of the levels present in E18 brain. (n = 3, * = p≤0.05, ** = p≤0.01, *** = p≤0.001).

Levels of total SUMO-1 conjugation in cerebrum remain relatively stable during development Levels of total SUMO-1 conjugation in cerebellum appear to increase slightly with age but the differences were not significantly different from E18 (Fig 3A). As noted, however, these values are very strongly influenced by the two major immunoreactive bands. SUMO-1 conjugation to the 100 kDa band in both cerebrum and cerebellum decreases rapidly around birth and then remains relatively constant at a much lower level (Fig 3B). In contrast, SUMOylation of the 35 kDa band remains stable and appears to increase with time (Fig 3C). Levels of SUMO-1 conjugation to all other proteins (i.e the entire lane minus the 100 and 35 kDa bands) appear variable. There is an initial decrease with age in the cerebrum (P1; 53.3% ± 5.3%) and remain relatively stable thereafter. Levels of total SUMO-1 conjugation in cerebellum decrease and remain significantly lower at all subsequent age points tested (Fig 3D).

**Fig 4:**
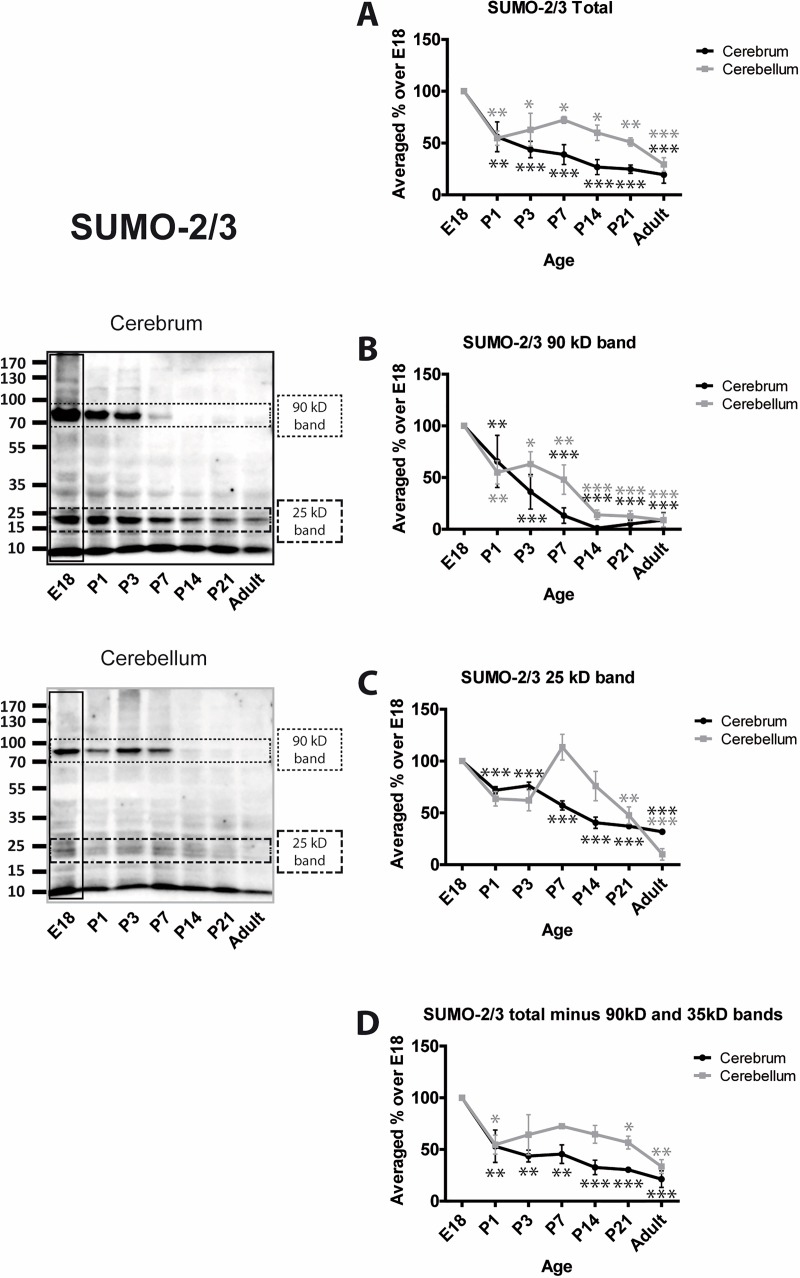
Levels of SUMO-2/3 conjugation to substrate proteins. Representative blots and quantification of the intensity of the whole SUMO-2/3 lane, the indicated bands and the whole lane minus the indicated bands. Total SUMO-2/3 immunoreactivity was quantified by taking the whole lanes (vertical black rectangle). Since the 90 kDa and 25 kDa bands are very intense and exhibit different profiles, they were subtracted from the total and the remaining signal plotted (Total (-) bands). This represents the level of SUMOylation of the other proteins in the sample. Graphs show the levels of immunoreactivity at different ages expressed as a percentage of the levels present in E18 brain. (n = 3, * = p≤0.05, ** = p≤0.01, *** = p≤0.001).

Using the 8A2 antibody the profile for total SUMO-2/3 conjugation decreases with age in both cerebrum and cerebellum. There is an initial decrease around birth to 53.1% ± 17.7% and 54.7% ± 9.2%, respectively, at P1 and 21.4% ± 8.0% and 33.5% ± 6.7% in adult (Fig 4A). SUMO-2/3 conjugation to the 90 kDa band displays a strong age-dependent decrease with little conjugation evident in cerebrum at P14 or thereafter and similar, but less pronounced, reductions in cerebellum (Fig 4B). SUMO-2/3-ylation of the 25 kDa band decreases steadily in cerebrum with age but in cerebellum the levels are not reduced by P14. However, in both brain regions SUMO-2/3 conjugation to this substrate is significantly decreased in P21 and adult. (Fig 4C). The profile of SUMO-2/3 conjugation to all other proteins (i.e. the entire lane minus the 90 and 25 kDa bands) almost exactly mirrors the total SUMO-2/3 (Fig 4D).

Interestingly, the differential trend between cerebrum and cerebellum of SUMO-2/3 conjugation resembles that of the SUMOylation machinery proteins, which was not the case for SUMO-1.

### Intracellular localisation of the SUMOylation machinery and SUMO substrates

**Fig 5:**
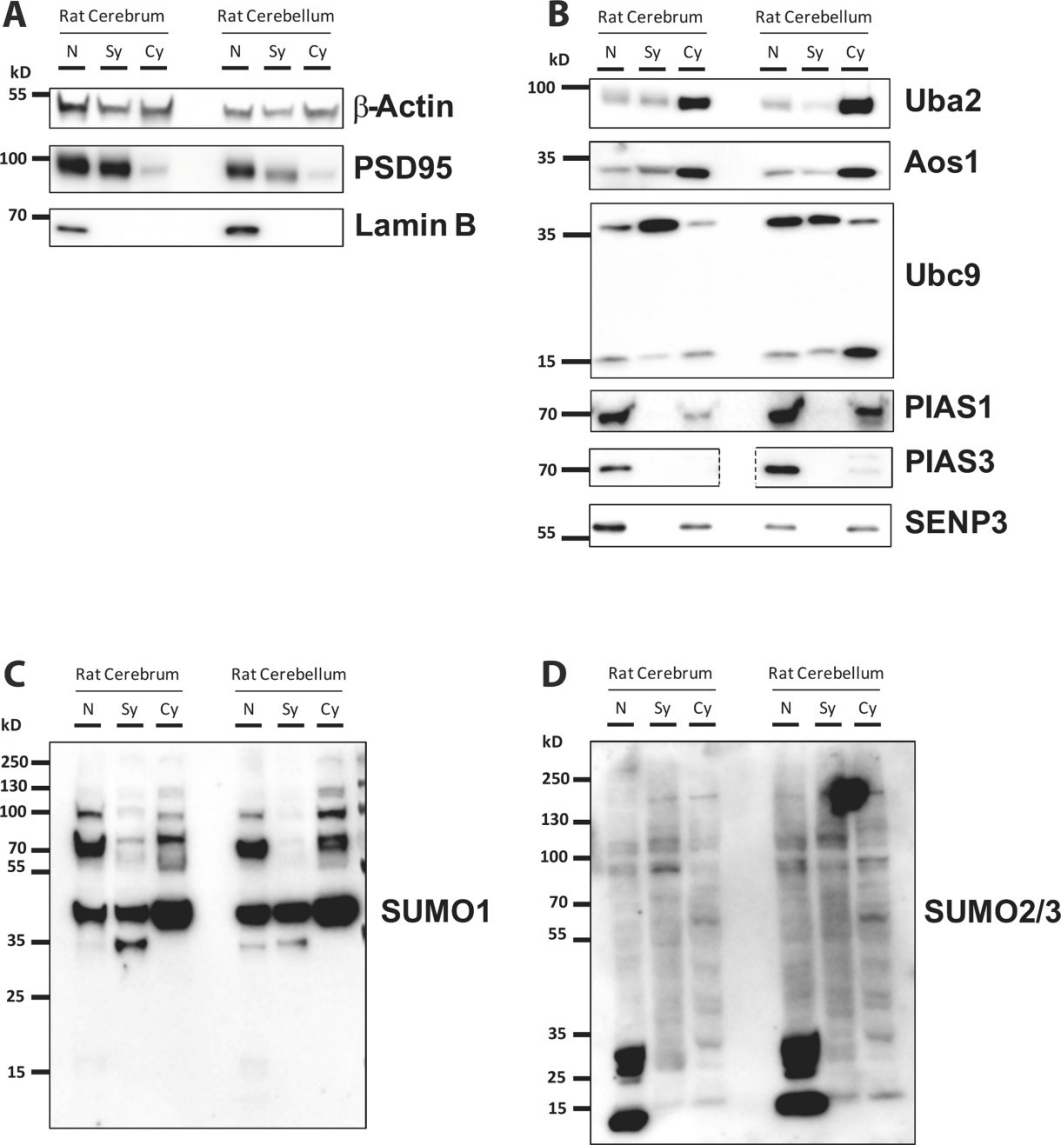
SUMO machinery and substrate proteins profiles in subcellular fractions. Representative blots of nuclear (N), synaptosomal (Sy) and cytosolic (Cy) fractions from adult rat cerebrum and cerebellum. 15μg of protein was loaded in each lane. A) fraction markers, B) SUMO machinery proteins, C) Proteins SUMOylated by SUMO-1, D) Proteins SUMOylated by SUMO-2/3.

To investigate the compartmentalisation of SUMO pathway proteins we prepared nuclear (N), synaptosomal (Sy) and cytosolic (Cy) fractions from adult cerebrum and cerebellum (Fig 5). The integrity of the fractions was determined by Western blotting for marker proteins. Lamin B and PSD95 were selected as markers for nucleus and synaptosomes, respectively. Each of these markers was enriched in their appropriate fraction (Fig 5A).

The E1 components Uba2 and Aos1 were present in all fractions but more abundant in cytosol in both cerebrum and cerebellum (Fig 5B). Ubc9 showed marked differences in the relative abundance of its unmodified and higher molecular weight SUMOylated form (Fig 5B). Interestingly, in cerebellum there was a reciprocal relationship between the bands with Ubc9 predominantly present in its modified form in the nucleus and synaptosomes but the unmodified form most prevalent in the cytosol. In cerebrum, the most abundant band was the modified form in synaptosmes. However, the differences in both forms of Ubc9 between nucleus and cytosol, was not as marked as in cerebellum. PIAS1 and PIAS3 were abundant in the nucleus but were not detected in the synaptosomal fraction. Both PIAS proteins showed higher expression levels in cerebellum than in cerebrum (Fig 5B). Finally, SENP3 was present in the nuclear and cytosolic fractions but not in the synaptosomes and its levels of expression was highest in the cerebrum nuclear fraction (Fig 5B). Strikingly, in cerebellum, it was almost equally portioned between nucleus and cytosol (Fig 5B). However, it is consistent with a role for SENP3 in the cytosol and at mitochondria [26, 45].

Proteins conjugated to SUMO-1 were present in all fractions, albeit at lower levels in the synaptosomes than in the other two fractions. The pattern of SUMO-1 substrates was similar between cerebrum and cerebellum although less signal was detected in synaptosomes prepared from cerebellum. Some differences were apparent between the cerebral and cerebellar nuclear and cytosolic fractions between those two regions (Fig 5C). Interestingly, SUMO-2/3 was present in all of the fractions and, in both regions, the synaptosomal fraction was labelled with approximately the same intensity as the nuclear and cytosolic fractions indicating a particular enrichment of SUMO-2/3 conjugation at synapses compared to SUMO-1. The most notable feature for SUMO-2/3 is the very intense immunoreactive bands between 10 and 35 kDa in the nuclear fraction. The lowest band likely corresponds to free SUMO2/3, which is much more abundant in the nucleus than in any other fraction (Fig 5D).

## Discussion

Taken together our results provide further evidence that SUMOylation machinery and SUMO conjugation in general is most active prior to and around birth in brain. Thereafter levels decrease with age. Our data broadly supports previous research by Loriol et al., 2012 [43] and later by Hasegawa et al. 2014 [46], which also observed a decrease in SUMOylation and SUMO pathway proteins throughout development. Moreover, we have extended these findings by analysing additional proteins from the SUMOylation machinery, such as the E1 enzyme Uba2, the deSUMOylating enzyme SENP3 and the E3 ligases PIAS 1 and PIAS3.

To validate our homogenates prior to investigating the SUMOylation machinery, we measured the developmental profiles of several key brain proteins. Importantly, our developmental profiles match previous studies for GluA1 [38, 47] NR1 [39–41] and PSD95 [36, 37], thus validating our experimental methodology and analysis (S2 Fig).

In contrast to PSD95 and syntaxin1A, which increase with age, all of the SUMO machinery proteins we investigated show a similar general pattern of decline during development in both cerebrum and cerebellum. Furthermore, the developmental pattern for SUMO conjugation is similar between cerebrum and cerebellum. However, it is important to take into account the fact that we are measuring the net signal from a complex mix of substrates. For individual proteins SUMOylation may increase, remain unchanged or decrease during development. This is exemplified by analysis of two prominent SUMO1 substrate bands at 100 kDa and 35 kDa, which display different conjugation profiles. It should also be noted that we are measuring levels under basal conditions, and the animals were not subjected to, for example, metabolic stress, which can radically alter protein SUMOylation.

While we were primarily interested in how the levels of SUMOylation and SUMO machinery proteins change within either cerebrum or cerebellum during development, the design of our experimental approach also allows us to make direct comparisons of the levels of proteins of interest between these two regions. Since, for each time point tested, each set of cerebrum and cerebellum samples were run against the same whole brain E18 homogenate, these E18 samples essentially act as an internal control allowing direct comparison of protein levels between cerebrum and cerebellum. Therefore, we have plotted the expression levels of the proteins of interest examined in cerebrum versus cerebellum in S3 and S4 Figs.

This analysis demonstrates that total SUMO1 conjugation levels in cerebrum and cerebellum do not differ significantly over development; however, the 100kDa band is slightly but significantly higher in cerebellum from P7 onwards. In contrast, total SUMO2/3 conjugation exhibits a peak at P7 in cerebellum that does not occur in cerebrum, largely due to a dramatic increase in abundance of the 25 kDa SUMO2/3-reactive band in cerebellum.

In terms of the SUMOylation machinery, we found that Uba2 and Aos1 expression in cerebellum rise above the levels in cerebrum from P3 and remain that way through adulthood. Furthermore, Ubc9, PIAS1, PIAS3 and SENP3 all experience a variable peak, occurring from P7 to P21, of significantly higher levels of expression in cerebellum than in cerebrum. Broadly speaking, these results indicate that the expression of the SUMOylation machinery is significantly higher in cerebellum than cerebrum throughout development. While these changes are not reflected in gross changes in total SUMO1 or SUMO2/3 conjugation in cerebellum versus cerebrum, this may be expected due to the higher levels of enzymes that both promote and counteract protein SUMOylation in cerebellum. Nonetheless, these findings suggest that SUMOylation may be more dynamically regulated in cerebellum than cerebrum during development.

Interestingly, we also observed that while the profile of Ubc9 + SUMO-Ubc9 is not significantly different through development in cerebrum or cerebellum, the level of Ubc9 increases in cerebellum by P14 and P21, compared to E18. Strikingly, the ratio of SUMO-Ubc9 to Ubc9 increases more during development in cerebrum than in cerebellum, being approximately 3-fold higher in cerebrum by the adult stage (S4 Fig). Thus, during adulthood cerebrum contains around 3 times more SUMOylated Ubc9 to Ubc9 than cerebellum. It has been previously shown in the literature that SUMOylation of Ubc9 modifies its target discrimination [42, 48, 49]. Moreover, the classical function of Ubc9 as an E2 may convert to an E3 ligase type of function when SUMOylated [50]. However, the implications of this finding in brain development and function are unknown, which could make this line of research an attractive target for investigation in the future.

Previous studies have investigated age- and activity-dependent changes in SUMOylation pathways in the brain [51–53]. Overall, their findings are consistent with our results, showing relatively high levels of SUMOylated substrates and SUMO machinery proteins early in development followed by an age-dependent decline. This reduction is consistent with the fact that during neuronal outgrowth and synapse development there is intense transcriptional and translational activity, processes in which SUMOylation is well-established to participate [54]. Similarly, SUMOylation is a key regulator of multiple protein interactions involved in synapse formation, stabilisation and function [3, 22]. There is intense activity in these processes during development, which decreases as neuronal architecture and connectivity develop and mature networks become established. The differences between the cerebrum and cerebellum may reflect the different influence of the SUMO pathway in cell type specificity, anatomical and functional characteristics, rates of maturation and in protein targets.

## Conclusion

In summary, in this work, our results describe a relatively consistent general developmental profile for the regulation of SUMO machinery proteins and SUMOylated substrates, with regional variations between cerebrum and cerebellum. This overall picture provides a clear context for future studies focused on how specific SUMO machinery proteins or defined SUMOylation substrate proteins in different CNS regions and cellular compartments are individually regulated during development.

## Supporting information

S1 Fig Developmental profiles of common marker proteins. The cytoskeletal proteins β-III-tubulin and β-actin, were monitored as general markers, commonly used as protein loading control. Representative immunoblots of cerebrum and cerebellum. Graphs show the levels of immunoreactivity at different ages expressed as a percentage of the levels present in E18 brain. (n = 3, * = p≤0.05, ** = p≤0.01, *** = p≤0.001).
(TIFF)

S2 Fig Developmental profiles of synaptic marker proteins. The postsynaptic protein PSD95, the presynaptic protein syntaxin1A and the AMPA and NMDA neurotransmitter receptor subunits GluA1 and NR1 (NMDAR1) were monitored as general markers of synapse formation. Representative immunoblots of cerebrum and cerebellum. Graphs show the levels of immunoreactivity at different ages expressed as a percentage of the levels present in E18 brain. (n = 3, * = p≤0.05, ** = p≤0.01, *** = p≤0.001).
(TIFF)

S3 Fig Differences between expression profiles in cerebellum and cerebrum of common and synaptic marker proteins. The cytoskeletal proteins β-III-tubulin and β-actin, and the postsynaptic protein PSD95, the presynaptic protein syntaxin1A and the AMPA and NMDA neurotransmitter receptor subunits GluA1 and NR1 (NMDAR1) were monitored in two brain regions over time. Their immunoreactivity profiles expressed as a percentage of the levels present in E18 brain in cerebellum vs. cerebrum were compared for each time point. (n = 3, * = p≤0.05, ** = p≤0.01, *** = p≤0.001).
(TIFF)

S4 Fig Differences between expression profiles in cerebellum and cerebrum of SUMOylation machinery proteins and SUMO1 and SUMO2/3 conjugated proteins. The SUMOylation machinery proteins Aos1, Uba2, Ubc9, PIAS1, PIAS3, SENP3 and SUMO1 and SUMO2/3 conjugated proteins were monitored in two brain regions over time. Their immunoreactivity profiles expressed as a percentage of the levels present in E18 brain in cerebellum vs. cerebrum were compared for each time point. (n = 3, * = p≤0.05, ** = p≤0.01, *** = p≤0.001).
(TIFF)

S1 Table Mean and standard error of the mean (SEM). This table includes the numerical data of the time courses performed for different proteins in cerebrum and cerebellum.
(XLSX)
