# Supplementary figures and images for: Developmental profiles of SUMOylation pathway proteins in rat cerebrum and cerebellum
Source: PLoS One. 2019 Feb 22;14(2):e0212857. doi: 10.1371/journal.pone.0212857 (PMC6386258; doi:10.1371/journal.pone.0212857)

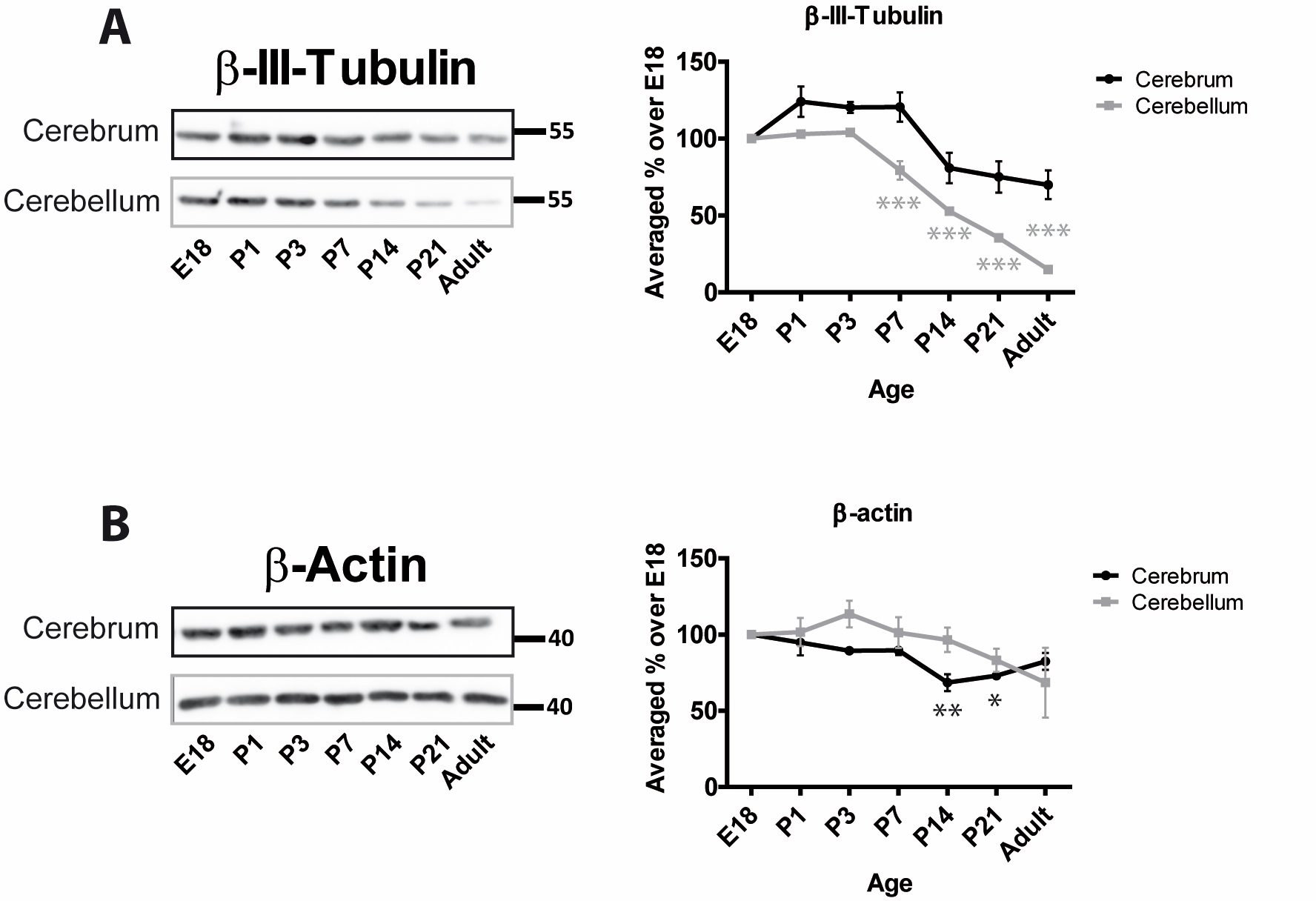

Supplement: S1 Fig — The cytoskeletal proteins β-III-tubulin and β-actin, were monitored as general markers, commonly used as protein loading control. Representative immunoblots of cerebrum and cerebellum. Graphs show the levels of immunoreactivity at different ages expressed as a percentage of the levels present in E18 brain. (n = 3, * = p≤0.05, ** = p≤0.01, *** = p≤0.001). (TIFF) [file pone.0212857.s001.tiff]

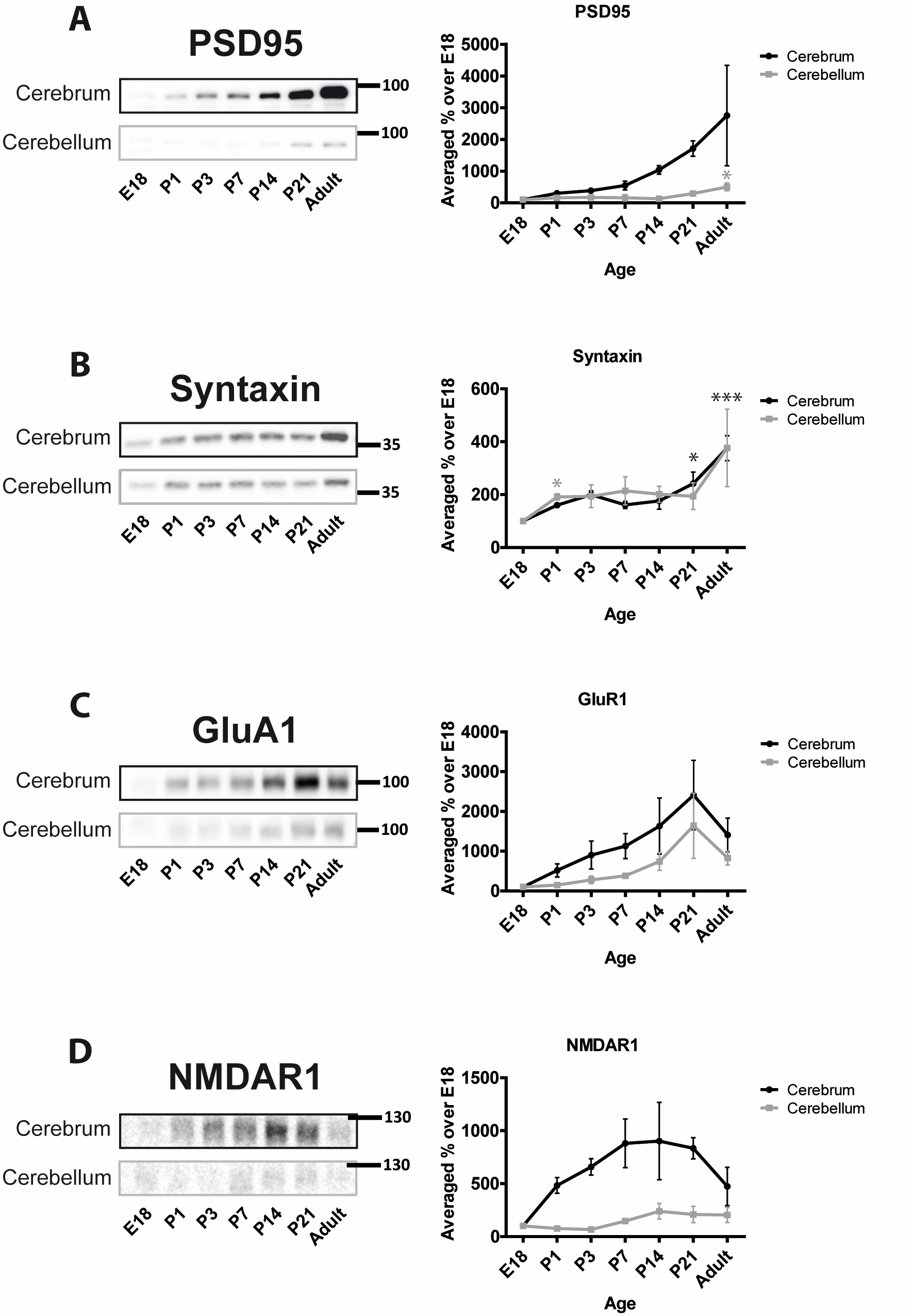

Supplement: S2 Fig — The postsynaptic protein PSD95, the presynaptic protein syntaxin1A and the AMPA and NMDA neurotransmitter receptor subunits GluA1 and NR1 (NMDAR1) were monitored as general markers of synapse formation. Representative immunoblots of cerebrum and cerebellum. Graphs show the levels of immunoreactivity at different ages expressed as a percentage of the levels present in E18 brain. (n = 3, * = p≤0.05, ** = p≤0.01, *** = p≤0.001). (TIFF) [file pone.0212857.s002.tiff]

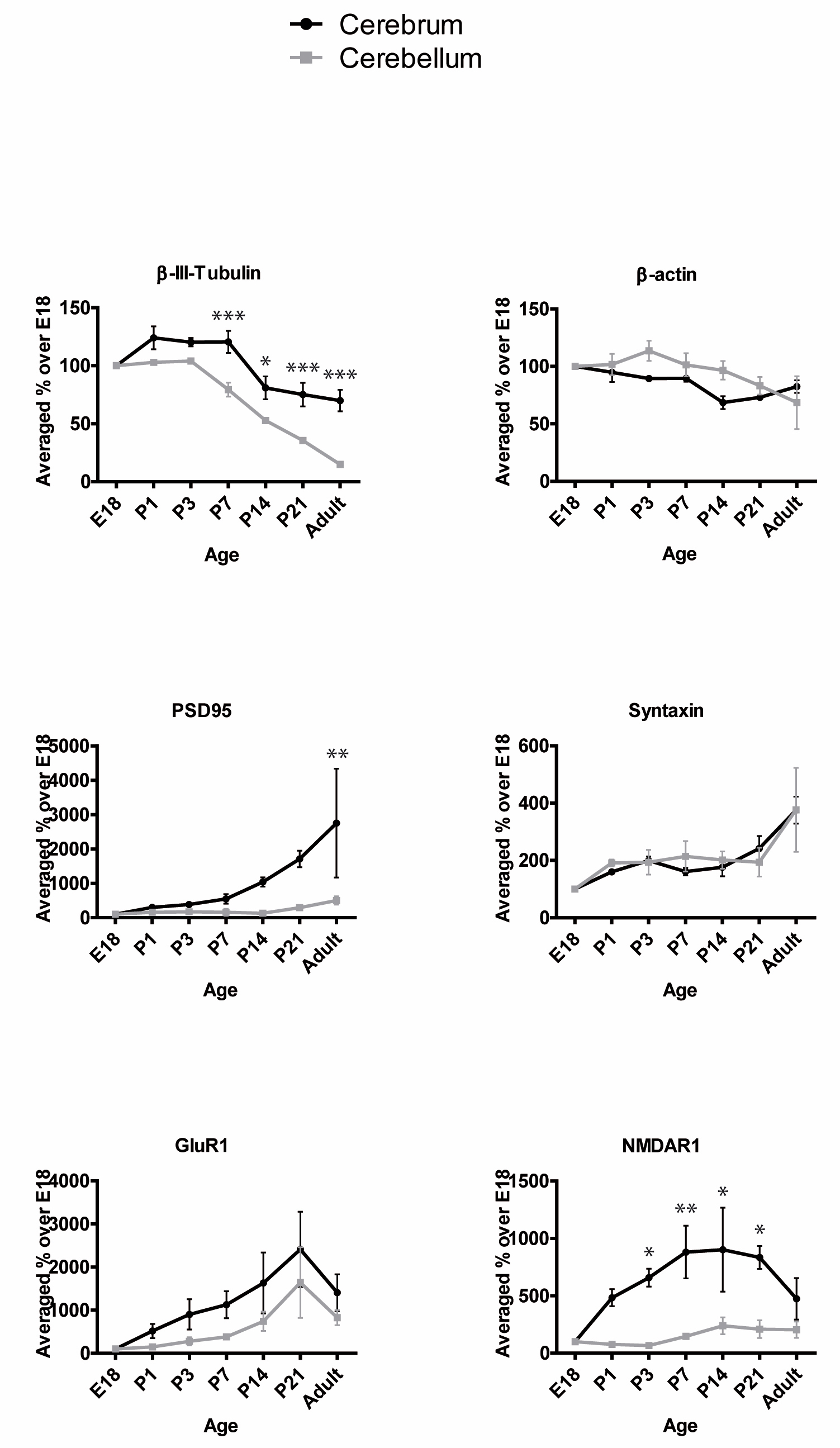

Supplement: S3 Fig — The cytoskeletal proteins β-III-tubulin and β-actin, and the postsynaptic protein PSD95, the presynaptic protein syntaxin1A and the AMPA and NMDA neurotransmitter receptor subunits GluA1 and NR1 (NMDAR1) were monitored in two brain regions over time. Their immunoreactivity profiles expressed as a percentage of the levels present in E18 brain in cerebellum vs. cerebrum were compared for each time point. (n = 3, * = p≤0.05, ** = p≤0.01, *** = p≤0.001). (TIFF) [file pone.0212857.s003.tiff]

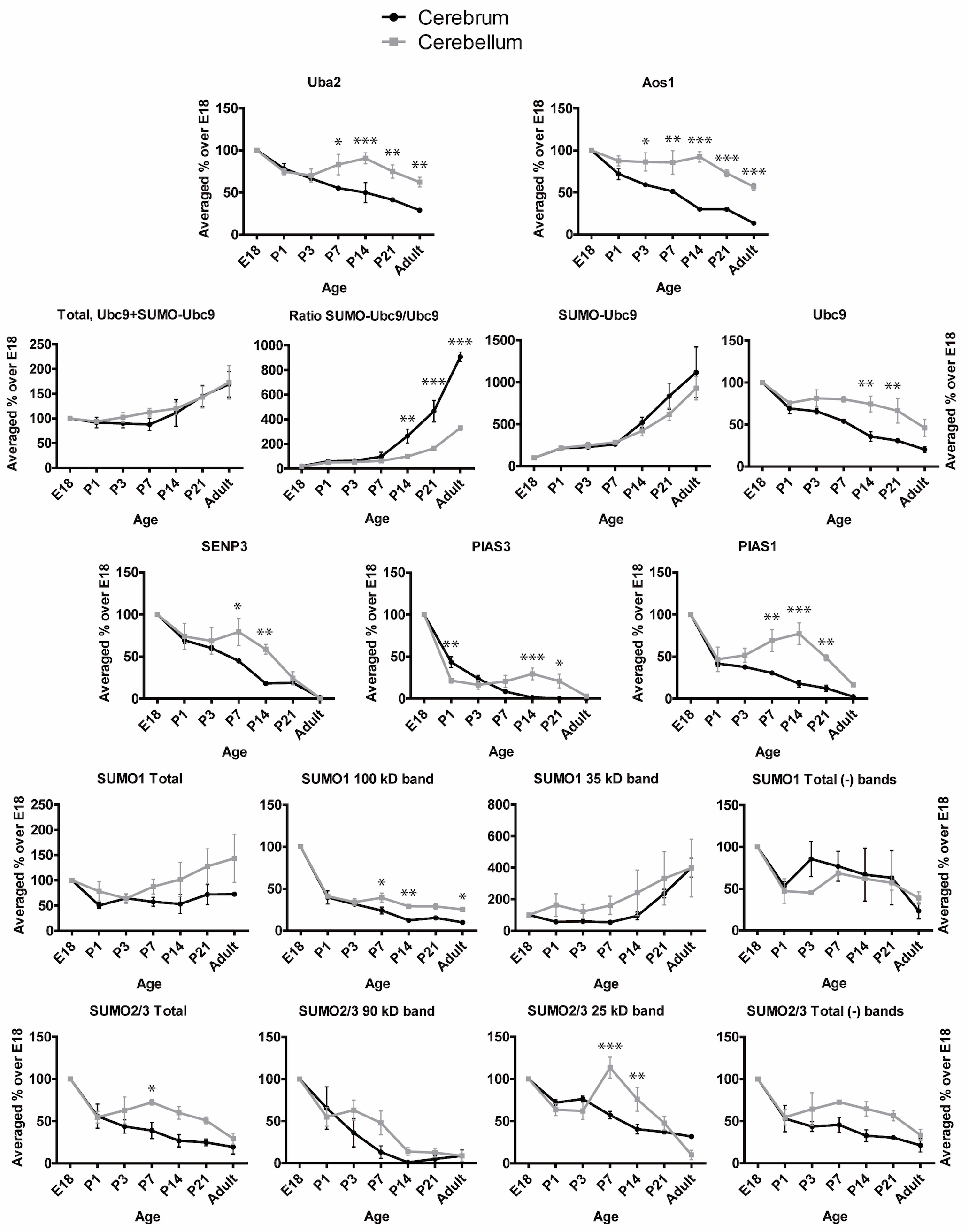

Supplement: S4 Fig — The SUMOylation machinery proteins Aos1, Uba2, Ubc9, PIAS1, PIAS3, SENP3 and SUMO1 and SUMO2/3 conjugated proteins were monitored in two brain regions over time. Their immunoreactivity profiles expressed as a percentage of the levels present in E18 brain in cerebellum vs. cerebrum were compared for each time point. (n = 3, * = p≤0.05, ** = p≤0.01, *** = p≤0.001). (TIFF) [file pone.0212857.s004.tiff]
